# Whether Urbanization Has Intensified the Spread of Infectious Diseases—Renewed Question by the COVID-19 Pandemic

**DOI:** 10.3389/fpubh.2021.699710

**Published:** 2021-11-24

**Authors:** Dongsheng Yu, Xiaoping Li, Juanjuan Yu, Xunpeng Shi, Pei Liu, Pu Tian

**Affiliations:** ^1^School of Economics, Zhongnan University of Economics and Law, Wuhan, China; ^2^Australia-China Relations Institute, University of Technology Sydney, Sydney, NSW, Australia; ^3^School of Economics, Zhengzhou University of Aeronautics, Zhengzhou, China

**Keywords:** population urbanization, land urbanization, infectious diseases, public health, GMM model

## Abstract

The outbreak of the COVID-19 epidemic has triggered adiscussion of the relationship between urbanization and the spread of infectious diseases. Namely, whether urbanization will exacerbate the spread of infectious diseases. Based on 31 provincial data from 2002 to 2018 in China, the impact of urbanization on the spread of infectious diseases from the dimensions of “population” and “land” is analyzed in this paper by using the GMM (generalized method of moments) model. The empirical study shows that the population increase brought by urbanization does not aggravate the spread of infectious diseases. On the contrary, urban education, employment and entrepreneurship, housing, medical and health care, and other basic public services brought by population urbanization can help reduce the risk of the spread of infectious diseases. The increasing density of buildings caused by land urbanization increases the risk of the spread of infectious diseases. Moreover, the impact of urbanization on the spread of infectious diseases has regional heterogeneity. Therefore, the prevention and control of disease play a crucial role.

## Introduction

Public health is not only related to the national economy and the livelihood of the people but also concerns national security and social stability (1), especially during the COVID-19 pandemic. Therefore, the research on urban public health is of great importance for both country and people. The outbreak of the COVID-19 epidemic has triggered the discussion of the relationship between urbanization and the spread of infectious diseases. One hypothesis attributes the intensified transmission of COVID-19 to the “urban diseases” caused by the rapid urban expansion, such as increased population ratio, dense building, environmental pollution, and deteriorated sanitation by many people, which finally threatens residents' public health. The main basis of this hypothesis is that there is a significant gap in the number of confirmed COVID-19 cases between urban and rural areas in various cities. Especially in Hubei Province, most of the confirmed COVID-19 cases appeared in central urban areas, such as the urban areas of Wuhan City as well as the downtown areas of Xiaogan and Huanggang, both of which are near Wuhan. On the contrary, the morbidity rate in rural areas in Hubei Province is relatively low. They also found that since the government locked down Wuhan, most of the new cases in Hubei also appeared in Wuhan's urban area.

The hypothesis owed the spread of COVID-19 to a “large population and too many buildings in big cities” and even claimed that the government should be restricting the population flow into large cities, evacuating urban populations, and reducing the building area, so as to prevent the spread of infectious diseases. Therefore, is the spread of Category B infectious diseases^1^ like SARS and COVID-19 really caused by a large population and building density in large cities? will urbanization exacerbate the spread of infectious diseases? In the context of the prevention and control of COVID-19, the answers to these questions have important reference value for correctly understanding and grasping urban public health safety, prevention, and treatment of infectious diseases, so as to propel the sustainable development of urbanization.

To explore the answers, we matched provincial data with health data to construct panel data in China's 31 provinces from 2002 to 2018, with a view to investigating the relationship between urbanization and the spread of infectious diseases.

The marginal contributions of this paper can be summarized as below. Firstly, the paper differentiates between population urbanization and land urbanization. “Population urbanization” is measured by the proportion of urban population, which is fundamentally different from “population density.” “Land urbanization” is measured by the proportion of built-up area, which can reflect the density of urban buildings to some extent. Secondly, the morbidity and mortality of Category A and B are used to reflect the spread of infectious diseases. The morbidity and mortality of Category A and B infectious diseases are significantly different from the population mortality (2), life expectancy per capita (3), newborn mortality (4), and prevalence of underlying diseases (5, 6) in the previous literatures. The former belongs to infectious diseases, which have the characteristics of “human-to-human transmission.” It is more easily to measure the spread of infectious diseases. The latter is mainly to measure life health, which is a composite indicator. But the mortality and the ultimate lifespan and prevalence of underlying diseases do not belong to infectious diseases, and the death and lifespan may be determined by the infectious diseases or by other reasons. Therefore, the former index is more accurate, scientific, and suitable for the subject. Moreover, this is more in line with the theme of this study.

The remainder of the analysis is organized as follows: the Literature Review section shows a review of related papers. The model construction and variable selection of urbanization and the spread of infectious diseases is shown in the section Model and Variables. The data is presented in the Data section. The empirical analysis is presented in the Empirical Results section. This paper's research is summarized in the Conclusions and Policy Advice section. The limitations of the study are presented in the section Limitation.

## Literature Review

The spread of infectious diseases is the key area of urban public health and the weak link in the development of urban public health in recent years. The outbreak of COVID-19 in 2019 sounded an alarm for urban public health and safety. Research on the spread of infectious diseases initially belongs to the field of medical research, because medicine is committed to the treatment of diseases, to ensure people's life and health. Some scholars discussed the spatial distribution of the avian influenza (H5N1) outbreak (7), the regional differences of AIDS (8), the epidemic trend of pertussis (9), the regional distribution of neonatal tetanus cases (5), the dengue outbreak (10, 11), the transmission of COVID-19 (12–20) and other class A and B infectious diseases. These literatures mostly study the causes, laws, epidemic trends, and medical measures of infectious diseases from the perspective of medicine. This paper mainly discusses the impact of urbanization on the spread of infectious diseases from the perspective of sociology.

Many scholars have studied the social factors behind the spread of infectious diseases. Among them, the level of medical facilities is the most direct factor affecting the spread of infectious diseases. Mody et al. (21) based on the data of nursing homes in Michigan, found that the level of medical facilities in nursing homes is inversely proportional to the risk of disease infection of the elderly: that is, the higher the level of medical facilities in nursing homes, the lower the risk of disease infection of the elderly. Toda et al. (22) investigated the fatal cases of infectious diseases among children in Japan's top three hospitals and found that strengthening the construction of medical facilities can significantly reduce the proportion of children who died of infection. However, the level of economic development affects the regional medical level and then has an impact on the spread of infectious diseases. Bai et al. (23) uses the panel data of 29 regions in China to explore the impact of EPU on medical expenditure and finds that EPU has a positive spatial spillover effect on medical expenditure. Su et al. (24) found that there was an inverted U-shaped correlation between economic growth and health, and the health promotion effect of economic growth decreased significantly when it exceeded the threshold.

In addition, aging is also an important factor affecting the spread of infectious diseases (25). Hence, 2019 novel coronavirus pneumonia is mostly found in the elderly, and the death cases are mostly elderly. This is mainly because as age increases, people over 45 years old will gradually show the characteristics preceding old age, such as slow metabolism, decreased resistance, decreased physiological function (26, 27), poor awareness of disease prevention, and become a susceptible and high-risk group for infectious diseases. Heravi et al. (28) collected cases of infectious diseases in 65 year old patients who were treated in a hospital in Turkey for the years 2010–2011. It was found that the elderly were susceptible to infectious diseases, and the incidence rate and mortality rate were generally higher. The incidence rate and mortaity rate of 45 notifiable infectious diseases in China were assessed by Yang et al. (29). It was found that the incidence and mortality of notifiable infectious diseases in the elderly population were significantly higher than that in young people in the period 2004–2013.

In recent years, many scholars found that air pollution has become an important factor affecting the spread of infectious diseases. Jang et al. (30) collected 660,000 infectious diseases data in Korea and studied the relationship between air pollution level and the incidence rate of notifiable infectious diseases in that country. It was found that the incidence rate of infectious diseases was highly correlated with air pollution. Mody et al. (21) found that respiratory diseases and the spread of infectious diseases are related to the level of air pollution. The higher the level of air pollution exposure, the higher the risk of respiratory diseases and the spread of infectious diseases. Maji et al. (31) and Zeng et al. (32) found a similar view when studying the impact of PM2.5 on the spread of infectious diseases. However, different from the previous literature, this paper focuses on the impact of urbanization on the spread of infectious diseases.

Urbanization, as an important factor discussed in this paper, has a controversial impact on the spread of infectious diseases. Some scholars believe that the promotion of urbanization has produced an agglomeration effect, which has brought “urban diseases” such as housing congestion, traffic congestion, environmental pollution and health deterioration (33, 34), and increased the risk to urban residents (26). The incidence rate and mortality rate of malaria in Africa were studied by Hay et al. (35), which quantified the malaria burden in Africa. The cities' accelerated urban lifestyle increased malaria incidence and mortality in Africa. Wu et al. (36) compared the differences of avian influenza outbreaks between developing and developed countries and found that the interaction of urbanization, income growth, and globalization exacerbated the spread of infectious diseases.

Other scholars believe that urbanization will increase residents' income (37), improve medical facilities and security systems (38), and improve medical standards and services (39), thereby reducing the incidence rate and mortality of infectious diseases (40). Neiderud (41) compared the development of economy, health, environment, infrastructure, and other social aspects between urban and rural areas, and found that the living conditions of the urban environment are generally better than that of the rural environment, and better housing, health, ventilation, and social services play a positive role in the prevention and control of infectious diseases.

In addition, the ability to monitor and control projects, and the effect of prevention and public knowledge projects or campaigns in cities is much better than that in rural areas, and they are more able to respond to sudden infectious diseases in a timely manner. Wood et al. (42) evaluated the spatial-temporal relationship of infectious disease data in 60 medium-sized countries and found that urbanization improved urban health and medical conditions, increased medical investment, and helped to reduce the burden of the spread of infectious diseases. Bauer et al. (43) found a similar view using population data and general practitioner practice data in England. These literatures mostly investigate the impact of urbanization on public health; there is a lack of research on the relationship between urbanization and the spread of infectious diseases, and few literatures study this problem from the perspective of heterogeneous urbanization. This paper studies these two aspects.

## Model and Variables

Based on the health production function proposed by Grossman (44) and referring to the method of Shao et al. (34), this paper integrates relevant factors affecting public health into the model and takes urbanization as an important factor affecting the spread of infectious diseases. The provincial health panel data of China's 31 provinces between 2002 and 2018 are used to empirically test the impact of urbanization on the spread of Category A and B infectious diseases. The empirical analysis model is expressed as follows:


(1) $$\begin{array}{l} {\text{lndisease}}_{it}=\alpha_{0}+\alpha_{1}{\text{lnurbanisation}}_{it}+\alpha_{2}\ln X_{it}+\mu_{it} \end{array}$$

where i represents China's 31 provinces and cities and t represents the year. disease reflects the spread of infectious diseases, urbanization represents the level of urbanization, *X*_*it*_ represents the control variable group, and μ is a random disturbance item. The indicators of each variable in the model are set as follows:

(1) **The explained variable**. This paper uses the morbidity and mortality of Category A and B legally reported infectious diseases to measure the spread of infectious diseases (disease). This is different from the population mortality (2), average life expectancy, and newborn mortality (3) in the previous literatures. The former belongs to the infectious diseases, which have the characteristics of “human-to-human transmission” and can better measure the spread of such infectious diseases. The lower the morbidity and mortality of Category A and B legally reported infectious diseases, the slower the spread of infectious diseases and the better the control.
  There are three specific reasons for selecting indicators of Category A and B infectious diseases for this paper. Firstly, COVID-19 has been classified as a Category B infectious disease. This paper is aimed to provide decision-making and reference for the prevention and control of COVID-19 through studying the previous data of Category A and B infectious diseases. Secondly, the morbidity and mortality of Category A and B infectious diseases are significantly different from the population mortality, average life expectancy, newborn mortality, and the prevalence of underlying diseases in the previous literatures. Thirdly, the indicators of Category A and B infectious diseases as used in this paper can also better respond to the hypothesis at the beginning of the article, which is also the author's original intention.
(2) **Core explaining variables**. Most of the literatures have used the ratio of urban population to total population in various regions to measure urbanization (2, 3). The indicator is fundamentally different from “population density,” as it reflects the increase of population proportion brought by the acceleration of the urban process, but population density refers to the number of people per unit area. This paper mainly discusses whether the population proportion increase brought by the urbanization process exacerbates the spread of infectious diseases. Therefore, the former index could yield a more accurate result. Although this measurement index conforms to the study that “urbanization leads to too many people” mentioned above, it does not involve the dimension that “urbanization leads to too dense buildings” above. Therefore, on the basis of the existing literatures, this paper measures urbanization not only by using the proportion of urban population to the total population from the dimension of the “population,” but also based on the proportion of the built-up area of a city to the total land area of the city area from the “land” dimension. To a certain extent, land urbanization can reflect the urban building density.
(3) **Control variables**. This paper uses the logarithm of the urban population density of different provinces and cities to measure population density, and compares it with the indicator of population urbanization, the main core explaining variable. In this paper, the dependency ratio of the elderly population in provinces and cities is used to measure the level of population aging (age) (3). Immunity is an important factor to resist infectious diseases. The elderly are worse than the young in terms of physical quality and physiological function (27), so their ability to fight against viruses is also naturally worse. The middle-aged and the elderly are more easily attacked by COVID-19, and most of the deaths are among the elderly. Moreover, in recent years, the problem of “getting old before getting rich” in China has constantly impacted regional economic growth, public health investment, and residents' medical consumption (45), which has a far-reaching impact on the health levels of residents. Per capita GDP (rgdp), which reflects the level of economic development and affects the income of residents, affects residents' health expenditure and health level (24), which affects the spread of infectious diseases. In this paper, the proportion of health expenditure to the total financial expenditure of provinces and cities is used to represent the public health input (expend), reflecting the importance of public health in the region. The medical development level (medical) is measured by the logarithm of the number of health technicians per 1,000 populations in cities, reflecting the regional medical construction level. The improvement of this indicator is conducive to the prevention, diagnosis, and treatment of infectious diseases. In this paper, the logarithm of the number of days with air quality reaching and better than level 2 in provinces and cities is used to measure the air quality level (air). Air is directly related to the spread of a great variety of respiratory diseases. Some of the pathogens can spread in the air freely, with the usual diameter of 5 microns. They can float on the surface dust in the air, floating in the air for a long time, and move for a long distance (46). The SARS virus in 2003 and COVID-19 in 2019 have the same principle. Therefore, air quality directly determines the speed and extent of disease spread.

## Data

This paper builds health panel data of China's 31 provinces between 2002 and 2018 by matching the provincial data and health data. All data studied in this paper are from *China Statistical Yearbook, China Health Statistical Yearbook, Statistical Yearbook of Provinces and Cities, National Economic and Social Development Statistical Publication*, National Research Network Database, and Guotai Junan database. The descriptive statistics of variables in the model are shown in Table 1.

**Table 1:** The descriptive statistics of variables.

**Variables**	**Observations**	**Mean**	**Standard**	**Min**	**Max**
Morbidity	527	5.495	0.357	4.513	6.604
Mortality	527	0.609	0.382	0.068	2.122
Population urbanization	527	0.499	0.154	0.005	0.896
Land urbanization	527	0.154	0.104	0.006	0.637
Population density	527	7.341	0.796	5.231	8.608
Age	527	0.125	0.027	0.067	0.219
Rgdp	527	10.211	0.777	8.089	11.851
Expend	527	0.061	0.019	0.027	0.106
Medical	527	1.542	0.359	0.693	2.738
Air	527	5.638	0.242	3.892	5.903

**Figure 1:**
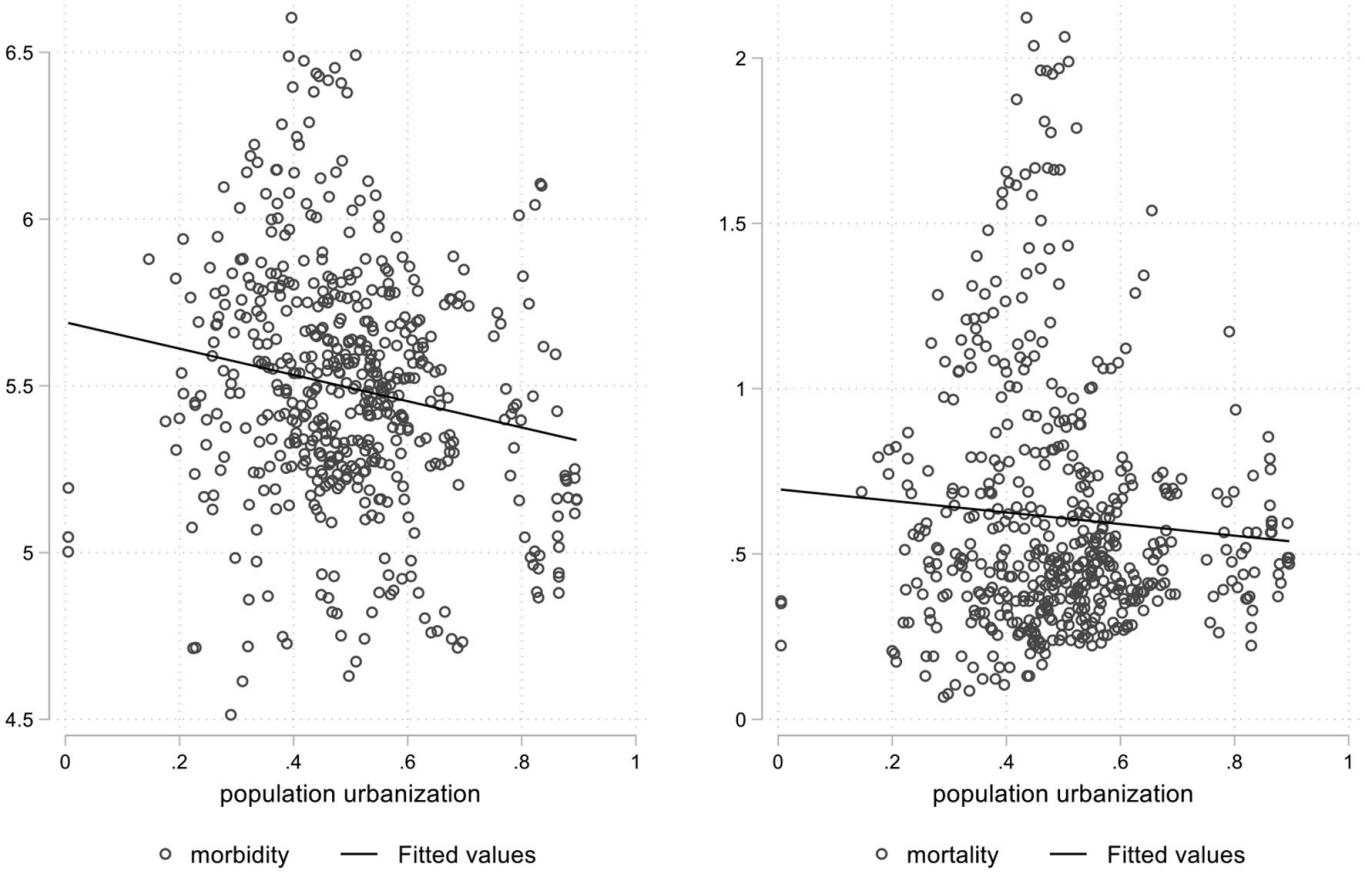
Fitting relationships between population urbanization and the morbidity and mortality.

**Figure 2:**
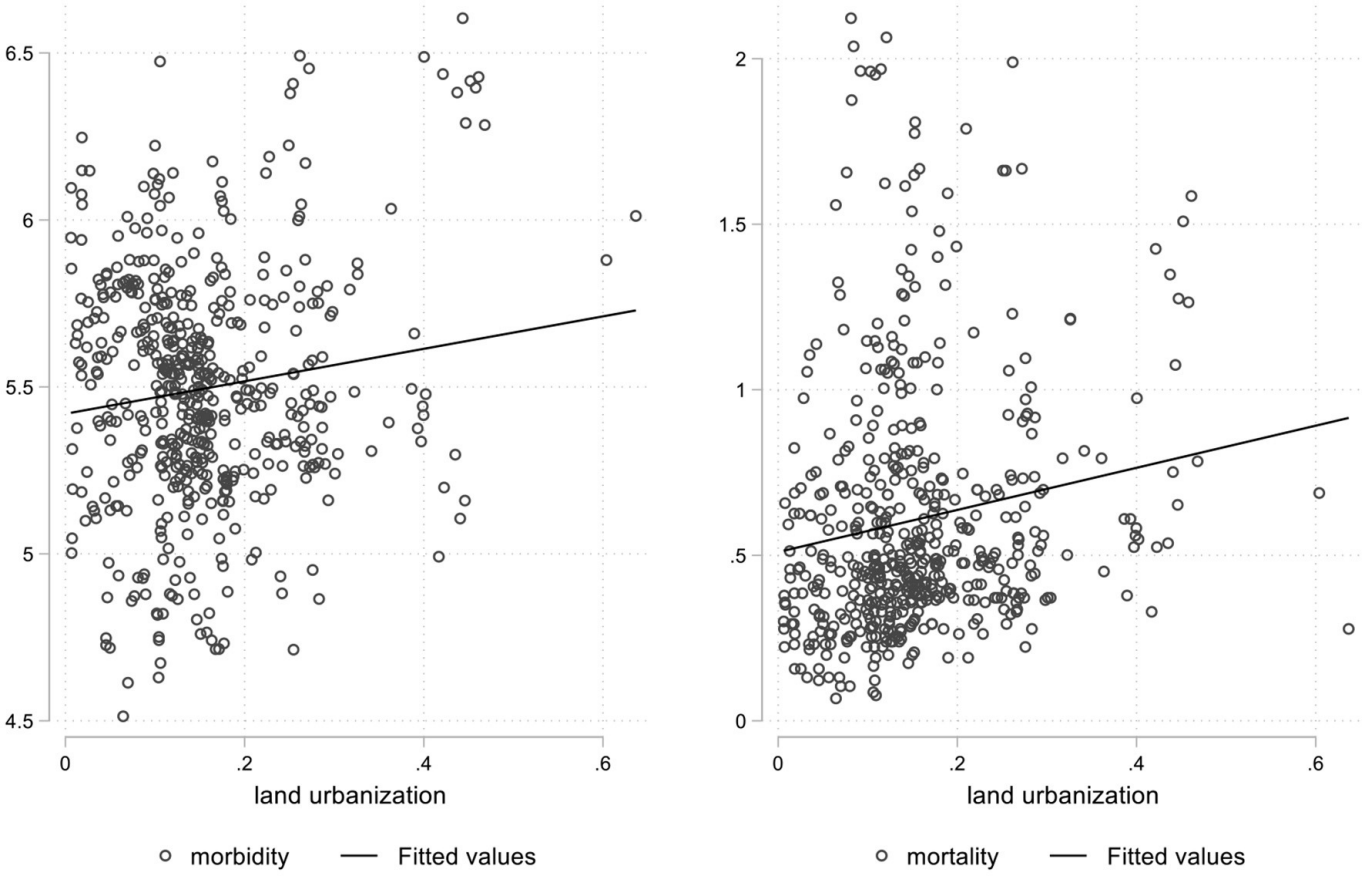
Fitting relationships between land urbanization and the morbidity and mortality.

To reflect the relationship between population urbanization, land urbanization, and the spread of infectious diseases more intuitively, this paper draws the figures for the fitting relationships between population urbanization and land urbanization and the morbidity and mortality of Category A and B infectious diseases, which is shown as Figures 1, 2, respectively. As can be seen from Figure 1, the level of population urbanization is significantly negatively correlated with the morbidity and mortality of Category A and B infectious diseases. Moreover, the degree of negative correlation between population urbanization and the morbidity of Category A and B infectious diseases is significantly higher than that of mortality, indicating that with the increase of population urbanization rate, the morbidity and mortality of Category A and B infectious diseases are decreasing. As can be seen from Figure 2, the level of land urbanization is significantly positively correlated with the morbidity and mortality of Category A and B infectious diseases, indicating that with the increase of land urbanization rate, the morbidity and mortality of Category A and B infectious diseases are on the rise. The correlations between population urbanization and land urbanization and the morbidity and mortality of A and B infectious diseases are completely opposite, and urbanization in different dimensions may vary in terms of the spread of infectious diseases. Therefore, for the purpose of testing the real causal relationship between population urbanization and land urbanization and morbidity and mortality of Category A and B infectious diseases, this paper will construct the provincial dynamic panel data GMM model in the next section for further empirical analysis.

## Empirical Results

The static panel estimation method is first adopted to estimate the panel data of China's 31 provinces between 2002 and 2018. The Hausman test for the endogeneity of explaining variables is also conducted. The *P* value of the Hausman test is 0.0043, rejecting the null hypothesis where all explaining variables are exogenous at the significance level of 1%. Therefore, on the basis of static panel regression, this paper adds the first-order lag term of the explained variable to construct the provincial dynamic panel data GMM model for estimation.^2^ To overcome the endogeneity in the model, generalized moment GMM is selected to estimate the full sample data.

The specific estimated results are shown in Table 2, where, Equations (1)–(4) represent the GMM estimation results of the system, while Equations (5)–(8) represent the difference GMM estimation results. *P* values provided by AR_2 of all the equations in Table 2 accept the null hypothesis at the significance level of 10%, indicating that the residual sequence of the difference equation in the model has only first-order sequence correlation and no second-order sequence correlation. The model has passed the autocorrelation test. The *P* values provided by Sargan test in Table 2 also accept the null hypothesis at the significance level of 10%, indicating that all instrumental variables are strictly exogenous and valid. Therefore, the estimation results of difference GMM and system GMM are consistent and reliable. The significance and direction of the regression coefficients of the core explaining variables and control variables in Table 2 for the morbidity and mortality of Category A and B infectious diseases are roughly the same, which further indicates that the empirical results are reliable.

**Table 2:** Baseline regression results of urbanization on the spread of infectious diseases.

**Variables**	**SYS-GMM**				**DIFF-GMM**			
	**(1)**	**(2)**	**(3)**	**(4)**	**(5)**	**(6)**	**(7)**	**(8)**
	**Morbidity**	**Mortality**	**Morbidity**	**Mortality**	**Morbidity**	**Mortality**	**Morbidity**	**Mortality**
Population urbanization	−0.359***	−0.174***			−0.364***	−0.060**		
	(−6.219)	(−2.987)			(−2.872)	(−2.013)		
Land urbanization			0.081**	0.070**			0.100**	0.089**
			(2.447)	(2.530)			(2.187)	(2.049)
Population density	5.617	2.223	6.839	2.603	2.608	7.713	3.413	7.823
	(1.548)	(1.603)	(1.255)	(1.077)	(1.038)	(1.213)	(1.461)	(1.131)
Age	1.119***	1.539***	0.958***	1.479***	1.330***	0.242	1.341***	0.017
	(3.095)	(6.776)	(3.416)	(6.268)	(4.208)	(0.965)	(4.121)	(0.062)
rgdp	−0.020	0.020	−0.064	0.007	0.021	0.199***	−0.009	0.213***
	(−0.741)	(1.362)	(−0.536)	(0.451)	(0.583)	(3.531)	(−0.344)	(3.839)
Expend	−0.504	−2.063***	0.179	−2.110***	−1.898***	−1.282***	−1.646***	−1.367***
	(−1.072)	(−6.220)	(0.556)	(−6.583)	(−3.291)	(−3.867)	(−3.115)	(−4.215)
Medical	0.043	−0.057***	0.018	−0.075***	−0.034	0.050	−0.083**	0.064
	(1.035)	(−2.850)	(0.553)	(−3.898)	(−0.820)	(1.284)	(−2.170)	(0.732)
Air	−0.026**	−0.005	−0.031**	−0.016	0.014	0.004	0.016	0.008
	(−2.072)	(−0.431)	(−2.346)	(−1.213)	(0.875)	(0.274)	(0.960)	(0.494)
L.disease	0.683***	0.776***	0.653***	0.797***	0.552***	0.287***	0.518***	0.314***
	(27.481)	(59.622)	(21.355)	(48.795)	(10.064)	(3.863)	(8.790)	(4.141)
AR_1	0.0069	0.0007	0.0065	0.0006	0.0092	0.0696	0.0081	0.0479
AR_2	0.6825	0.7024	0.5544	0.7616	0.8279	0.5552	0.7203	0.4926
Sargan text	0.4797	0.4137	0.4581	0.4475	0.459	0.4177	0.4278	0.433
Observations	527	527	527	527	527	527	527	527

***, **, *The significance level of 1, 5 and 10%, respectively. The numbers with asterisks in the corresponding rows of all variables in the table represent the estimation coefficients, and the values in the parentheses are the corresponding T statistical values, and the P values of AR_1, AR_2, and Sargan test are provided respectively.

The results of both system GMM and differential GMM regression show that population urbanization has a significant negative impact on the morbidity and mortality of Category A and B infectious diseases, while land urbanization has a significant positive impact on the morbidity and mortality of both above. Taking the empirical results of system GMM as an example, the morbidity and mortality will be reduced by 0.359 and 0.174%, respectively, with a 1% increase in population urbanization. The morbidity and mortality will be increased by 0.081 and 0.07%, respectively, with a 1% increase in land urbanization. This indicates that the improvement of population urbanization is conducive to reducing the morbidity and mortality of Category A and B infectious diseases, while the improvement of land urbanization increases the morbidity and mortality of both above.

Population urbanization does not aggravate the spread of infectious diseases. This is because people rush to big cities to secure more job opportunities, better job welfare, higher incomes, more favorable education and health care services, etc. (47), which can be summarized as “the people's aspiration for a better life” as called by President Xi. The development of urbanization essentially lies in “humans,” and “human needs.” The increasing urban population has brought the development of urbanization, which contributes to the economic effect, income effect, scale effect, rich medical and health resources, improves the demand for health, and forms health consciousness, etc. (36–39). These channels all reduce the risk of the spread of infectious diseases.

Land urbanization increases the risk of the spread of infectious diseases. This is because land urbanization certainly reflects the degree of “urban building density,” including the density of urban building land, industrial land, construction area, etc., which can directly affect the living environment, air quality, and health status of urban residents. With the enhancement of land urbanization, industrial building area, environmental pollution, and the decrease of ecological green space (48), the living environment and breathing air quality of citizens continuously deteriorate, which makes it possible to spread infectious diseases.

From the perspective of control variables, population density has no significant effect on the morbidity and mortality of Category A and B infectious diseases, indicating that the spread of infectious diseases has little relationship with population density. In the spread of infectious diseases, population density refers to the density in the sense of clustering. Even if you live in rural areas and the overall population density is not high, the disease will still spread if you live in groups. On the other hand, in areas with high population density in large cities, if efforts are made to avoid clustering and contact between people, there will be no transmission. So, disease prevention and control play a crucial role. Population aging has a significant positive impact on the morbidity and mortality of Category A and B infectious diseases, and the increase in the level of aging significantly increases the morbidity and mortality of Category A and B infectious diseases, exacerbating the spread of infectious diseases. The reason may be that, compared with the young, the elderly have weaker constitution and a lower awareness of disease prevention, making them vulnerable and the high-risk groups for the spread of infectious diseases. This is consistent with the views of scholars Song and Yang (49). This is also the reason why the morbidity and mortality of COVID-19 are mainly among the middle-aged and the elderly. Therefore, attention shall be fully paid to the population aging. Per capita GDP has different influences on the morbidity and mortality of Category A and B infectious diseases but has no influence on the spread of infectious diseases. However, in the differential GMM model, per capita GDP has a positive influence on the mortality of Category A and B infectious diseases. The reason is probably that the level of economic development increase is coupled with more serious environmental pollution, which is harmful to residents' health, induces diseases, increases the economic burden, and damages the labor ability. Thus, people will fall into poverty and be unable to bear the corresponding medical costs, eventually lost in the “poverty trap” of environmental health (50) and increasing the risk of death from infectious diseases. Level of investment in public health, medical development, and air quality on morbidity and mortality of Category A and B infectious diseases have significant negative effects, which suggests that the increase of the public health investment, medical development, and the improvement of air quality significantly reduce the morbidity and mortality of Category A and B infectious diseases, inhibiting the spread of infectious diseases.

The existing literature studies have shown that regions vary largely in terms of economic development, medical facilities, degree of aged individuals, and urbanization, leading to the obvious imbalance of health levels in different regions of China (1). To investigate the regional differences of the impact of Category A and B infectious diseases on urbanization, this paper divides all the samples into three regions, namely, the east, central and west parts, and tests the impact from the dimensions of population urbanization and land urbanization, respectively. Table 3 shows the estimated results of regional differences in the impact of population urbanization on Category A and B infectious diseases under the systematic GMM model.

**Table 3:** Regional differences in the impact of population urbanization.

**Variables**	**SYS-GMM**					
	**Eastern region**		**Central regions**		**Western regions**	
	**(1)**	**(2)**	**(3)**	**(4)**	**(5)**	**(6)**
	**Morbidity**	**Mortality**	**Morbidity**	**Mortality**	**Morbidity**	**Mortality**
Population urbanization	−0.6308*	−0.2719**	−0.3562	−0.4020	−0.3042	−0.6780
	(−1.957)	(−2.316)	(−0.086)	(−1.216)	(−1.446)	(−0.909)
Population density	3.1638	−7.4401	−6.6646	−8.8899	5.5022	2.4767
	(1.245)	(−1.232)	(−0.905)	(−0.910)	(0.778)	(0.395)
Age	0.8588	1.0626	2.7547**	1.0209	0.1665	0.6464*
	(1.101)	(0.182)	(2.035)	(1.178)	(0.017)	(1.845)
rgdp	0.2820	−0.1240	0.6602*	−1.2548	−1.1821**	−0.9506
	(1.061)	(−0.519)	(1.799)	(−0.706)	(−2.268)	(−1.308)
Expend	−1.0228*	−0.3583	−1.1218	1.9069	−1.9507*	0.7748
	(−1.836)	(−0.156)	(−1.549)	(1.191)	(−1.720)	(0.671)
Medical	−0.2009**	−0.4367**	−0.3246*	−0.4469	−0.1684	−0.4895
	(−2.225)	(2.138)	(−0.493)	(−1.078)	(−0.725)	(−0.396)
Air	−0.0461	−0.0440	−1.1638**	−0.3371	−1.0795	−0.1793
	(−1.294)	(−0.640)	(−2.114)	(−1.074)	(−1.472)	(−1.494)
L.disease	0.6021***	0.2870***	−1.2830**	−1.1777***	−1.0159**	3.4958**
	(6.033)	(3.038)	(−2.503)	(−2.976)	(−2.329)	(2.537)
AR_1	0.0644	0.0221	0.0189	0.0168	0.0822	0.0243
AR_2	0.4952	0.6207	0.349	0.9265	0.7641	0.4104
Sargan text	0.9998	1.0000	1.0000	1.0000	1.0000	1.0000
Observations	204	204	153	153	170	170

***, **, *The significance level of 1, 5 and 10%, respectively. The numbers with asterisks in the corresponding rows of all variables in the table represent the estimation coefficients, and the values in the parentheses are the corresponding T statistical values, and the P values of AR_1, AR_2, and Sargan test are provided respectively.

As indicated by the results in Table 3, the *P* values provided by AR_2 and Sargan tests both verify the null hypothesis at the significance level of 10%, which further proves the reliability of the regression results. Population urbanization in eastern China has a significant negative impact on the morbidity and mortality of Category A and B infectious diseases, indicating that the improvement of population urbanization in eastern regions is conducive to reducing the risk of infectious disease spread, which is consistent with the results of the full sample estimation. The impact of population urbanization on the morbidity and mortality of Category A and B infectious diseases in the central and western regions is negative but insignificant. The positive effect of population urbanization on the prevention and control of infectious diseases in the central and western regions has not been shown. The main reason may be that, compared with the central and western regions, the eastern regions have relatively developed health levels, abundant education and medical resources, relatively intact public health systems, a high education level and income of residents, and more rigorous requirements for living environment and health. All of the above will indirectly improve the quality of life of the inhabitants, the prevention and control of infectious diseases, and health consciousness. The most important thing is that the residents' needs for higher material and cultural levels and a better and healthy life in the eastern region can be satisfied much faster than that of the population gathering in the eastern region. Therefore, the urbanization of the eastern region has a more negative impact on the spread of infectious diseases.

Table 4 shows the estimated results of regional differences in the impact of land urbanization on the spread of Category A and B infectious diseases under the system GMM model. It can be seen from Table 4 that land urbanization in the eastern region has a significant negative impact on the morbidity of Category A and B infectious diseases, which reveals that the increase in land urbanization in the eastern region can help reduce the risk of infectious disease spread. It is not consistent with the estimated results. The impact of land urbanization on the morbidity and mortality of Category A and B infectious diseases in the central and western regions is significantly positive, indicating that the improvement of land urbanization in the western regions increases the risk of the spread of infectious diseases. The cause of the regional differences may be as follows:

**Table 4:** Regional differences in the impact of land urbanization.

**Variables**	**SYS-GMM**					
	**Eastern region**		**Central regions**		**Western regions**	
	**(1)**	**(2)**	**(3)**	**(4)**	**(5)**	**(6)**
	**Morbidity**	**Mortality**	**Morbidity**	**Morbidity**	**Mortality**	**Morbidity**
Land urbanization	−0.9601***	−0.4067	0.6192**	0.7437*	0.0177**	0.1575**
	(−2.880)	(−0.597)	(2.326)	(1.829)	(2.017)	(2.349)
Population density	3.3630	−1.1657	−3.3557	−2.0959	4.2387	7.3419
	(1.166)	(−0.928)	(−0.480)	(−0.483)	(0.186)	(1.182)
Age	0.8765	0.3759	2.5570**	−6.1144	3.2429	1.3849**
	(0.969)	(0.053)	(2.284)	(−0.516)	(0.100)	(2.347)
rgdp	0.1287	−0.1024	0.6453	0.4276	−0.7296	−1.8042
	(0.721)	(−0.709)	(1.628)	(0.641)	(−0.164)	(−1.470)
Expend	−0.1328**	−1.2658	−1.0627*	−1.5019	−1.2157	1.8885**
	(−2.218)	(−0.316)	(−1.810)	(−0.409)	(−0.211)	(2.083)
Medical	−0.4360**	0.0076	−5.6163*	1.2861	2.4147	0.5866
	(−2.190)	(0.099)	(−1.930)	(0.515)	(0.157)	(0.533)
Air	−0.0752**	−0.0040	0.5108	0.0017	−0.2058	−0.1424
	(−2.090)	(−0.077)	(0.982)	(0.017)	(−0.101)	(−0.405)
L.disease	0.5941***	0.4867***	−1.1119***	1.4820***	−0.1200***	3.2418**
	(5.983)	(3.199)	(−3.330)	(2.994)	(−4.053)	(2.323)
AR_1	0.0374	0.015	0.0163	0.0146	0.0662	0.0209
AR_2	0.4325	0.9854	0.2254	0.2385	0.7247	0.5320
Sargan text	0.9998	1.0000	1.0000	1.0000	1.0000	1.0000
Observations	204	204	153	153	170	170

***, **, *The significance level of 1, 5 and 10%, respectively. The numbers with asterisks in the corresponding rows of all variables in the table represent the estimation coefficients, and the values in the parentheses are the corresponding T statistical values, and the P values of AR_1, AR_2, and Sargan test are provided respectively.

In the process of urbanization, the eastern region adheres to the industrial transformation with the industrial structure level higher than that of the central and western regions. It strengthens the intensive utilization of land, protects the ecological environment of the cities, improves the urban living environment, and highlights green and healthy urbanization (48), so as to produce a positive “interactive” relationship between the land urbanization rate and infectious diseases prevention and control. Compared with the eastern region, the central and western regions have a lower level of economic development and threshold for environmental regulation. They undertake the transfer of some polluting industries in the eastern region and face the threat of pollution transfer from the eastern region (51). The easily destroyed ecological environment, the increased environmental pollution, and the deteriorated urban living environment and air quality have augmented the risk of the spread of infectious diseases (52).

To test the conclusion robustness of this study, the differential GMM model is used to estimate the regional heterogeneity of population urbanization, land urbanization, and the spread of infectious diseases. The specific regression results are shown in Tables 5, 6. To be specific, Tables 5, 6 show the robustness test results of the impact of population urbanization and land urbanization on the spread of infectious diseases, respectively. According to the regression results in Tables 5, 6, the direction and significance of the regression coefficients of population urbanization and land urbanization on the morbidity and mortality of Category A and B infectious diseases are roughly the same as those in Tables 3, 4, except for coefficients which are different. It further proves that the research conclusions of this paper are robust and reliable.

**Table 5:** The robustness test of population urbanization.

**Variables**	**DIFF-GMM**					
	**Eastern region**		**Central regions**		**Western regions**	
	**(1)**	**(2)**	**(3)**	**(4)**	**(5)**	**(6)**
	**Morbidity**	**Mortality**	**Morbidity**	**Morbidity**	**Mortality**	**Morbidity**
Population urbanization	−1.0352*	−2.1906*	−0.8034	−1.4020	−1.1195*	−0.4863
	(−1.853)	(−1.755)	(−1.062)	(−1.259)	(−1.686)	(−0.107)
Population density	1.9029	−5.1416	1.6359	−2.8899	2.4529	6.5785
	(1.642)	(−1.319)	(1.549)	(−0.946)	(1.589)	(1.387)
Age	1.1913	3.1623**	0.2904	57.0209	2.2026	−0.3496
	(1.315)	(1.966)	(0.038)	(1.216)	(0.270)	(−0.103)
rgdp	0.4467**	0.7777*	1.6158**	−1.2548	−0.9862	−0.4307
	(2.026)	(1.689)	(2.097)	(−0.725)	(−1.072)	(−1.175)
Expend	−2.7409	−0.3739	0.8189	1.9069	−0.4761	1.2627
	(−1.128)	(−1.318)	(0.795)	(1.246)	(−1.621)	(1.418)
Medical	−0.3118***	0.0167	−1.8586	−3.4469	8.0402	2.3041
	(−3.072)	(0.200)	(−0.804)	(−1.130)	(1.494)	(1.243)
Air	−0.0220	−0.0677*	−0.5670	0.3371	−0.9307	−0.1791
	(−0.618)	(−1.688)	(−1.074)	(1.121)	(−1.349)	(−1.087)
L.disease	0.5536***	−1.2917**	0.5408***	−1.1777***	−0.9155***	−0.1776***
	(5.062)	(−2.067)	(2.912)	(−3.037)	(−3.321)	(−4.259)
AR_1	0.0519	0.0364	0.0217	0.0926	0.0866	0.0658
AR_2	0.528	0.518	0.1518	0.1422	0.2762	0.2693
Sargan text	0.8934	0.9953	1.0000	1.0000	1.0000	1.0000
Observations	204	204	153	153	170	170

***, **, *The significance level of 1, 5 and 10%, respectively. The numbers with asterisks in the corresponding rows of all variables in the table represent the estimation coefficients, and the values in the parentheses are the corresponding T statistical values, and the P values of AR_1, AR_2, and Sargan test are provided respectively.

**Table 6:** The robustness test of land urbanization.

**Variables**	**DIFF-GMM**					
	**Eastern region**		**Central regions**		**Western regions**	
	**(1)**	**(2)**	**(3)**	**(4)**	**(5)**	**(6)**
	**Morbidity**	**Mortality**	**Morbidity**	**Morbidity**	**Mortality**	**Morbidity**
Land urbanization	−0.9252***	−1.6577	0.6192**	0.7437	0.1210***	0.0378**
	(−2.776)	(−0.768)	(2.402)	(1.339)	(2.934)	(2.390)
Population density	8.7626	2.0615	−3.3557	−2.0959	6.8670	3.3015
	(0.950)	(0.405)	(−0.544)	(−0.543)	(1.071)	(0.709)
Age	1.0876	−1.0846	2.5570**	−6.1144	1.3447	7.4087*
	(0.769)	(−0.640)	(2.474)	(−0.590)	(1.599)	(1.862)
Rgdp	0.1413	0.3092	0.6453*	0.4276	−0.7101**	−0.9036
	(1.047)	(0.975)	(1.825)	(0.716)	(−2.277)	(−1.019)
Expend	−0.2799	−2.8727	−1.0627**	−1.5019	−1.1651	1.3852
	(−0.087)	(−0.461)	(−2.043)	(−0.468)	(−1.456)	(1.635)
Medical	−0.2358**	0.1492*	−0.6163**	1.2861	0.1989	0.1083
	(−2.178)	(1.819)	(−2.133)	(0.579)	(1.007)	(0.156)
Air	−0.1463	0.0500	0.5108	0.0017	−0.1499	−0.0555
	(−0.969)	(1.297)	(1.103)	(0.017)	(−1.055)	(−0.228)
L.disease	0.5321***	−0.8979***	−1.1119***	1.4820***	−0.3722***	1.8112***
	(4.658)	(−2.998)	(−3.528)	(3.098)	(−2.949)	(3.643)
AR_1	0.0267	0.018	0.0407	0.0131	0.0162	0.0129
AR_2	0.1346	0.5454	0.204	0.1463	0.2434	0.4116
Sargan text	0.7656	0.8976	1.0000	1.0000	0.9995	0.9683
Observations	204	204	153	153	170	170

***, **, *The significance level of 1, 5 and 10%, respectively. The numbers with asterisks in the corresponding rows of all variables in the table represent the estimation coefficients, and the values in the parentheses are the corresponding T statistical values, and the P values of AR_1, AR_2, and Sargan test are provided respectively.

## Conclusion and Policy Advice

The COVID-19 pandemic renewed a question of whether the increase in population and the dense construction caused by urbanization increases the spread of infectious disease? To explore the relationship between urbanization and the spread of infectious diseases, this paper matches provincial data and health data to construct the panel data of China's 31 provinces between 2002 and 2018. Also, a GMM model is used to empirically evaluate the impact of urbanization on the morbidity and mortality of Category A and B infectious diseases from the dimensions of “population” and “land.” Findings are listed as below:

Firstly, the full sample regression results show that population urbanization and land urbanization have opposite effects on the morbidity and mortality of Category A and B infectious diseases. Higher population urbanization reduces the morbidity and mortality of Category A and B infectious diseases and inhibits the spread of infectious diseases. On the contrary, a higher land urbanization rate increases the morbidity and mortality of Category A and B infectious diseases and intensifies the spread of Category A and B infectious diseases.

Secondly, according to the results of regional heterogeneity regression, due to the developed medical level, rich educational and medical resources, public health system, and high quality of living environment and health requirements in eastern regions, the negative impact of population urbanization level on the spread of infectious diseases in eastern regions is more obvious than that in central and western regions.

Thirdly, population density has no obvious impact on the spread of infectious diseases so disease prevention and control play a crucial role. To a certain extent, the increase of the aging population and per capita GDP enhances the risk of the spread of infectious diseases. The enhancement of investment in public health, medical development, and air quality make the spread of infectious diseases less risky.

The policy inspirations of the conclusion in this paper mainly involve the following several aspects:

Firstly, China should continue to promote the “people-oriented” new urbanization construction and expand the positive effects of population urbanization on the prevention and control of infectious diseases and public health. In the face of the increasing proportion of urban population brought by urbanization, the government should not limit the inflow of population but improve the level of urban technology and management by adjusting production and lifestyle, so that people's demands for a better and healthier life can be met faster than population growth. The reform of the household registration system is particularly important, so efforts should be made to actively promote the adjustment and improvement of the points-based household registration policy in megacities and supercities. A mechanism has been established to link the basic public services such as urban education, employment and entrepreneurship, and medical and health care to the permanent population, so as to avoid the influence of population mobility on the epidemic spread. In this way, China can actively cope with the challenges to the prevention and control of infectious diseases and public health brought by the increase of urban population.

Secondly, China should have the consciousness of “safety blank” for the urban development, that is to optimize the urban space layout without blind expansion and excessive land development, so as to make the production space more intensive and efficient. Protecting the urban ecological and living environment can make land urbanization bring a positive “interactive” relationship with the prevention and control of infectious diseases and public health, thus maximizing the sustainability of urban development.

Thirdly, China should build infrastructure services such as health care, education, and old-age care that are compatible with urbanization, and improve the public health management system and the “diversified” old-age security system. In this way, the negative effects of urbanization on the public health of residents can be reduced, and high-quality public resources can better serve local residents, so as to deal with public health emergencies such as the outbreak of infectious diseases more calmly.

Fourthly, when formulating the policies with regard to the prevention and control of infectious diseases and public health, the government should consider the impact of regional differences and the local realities. In particular, efforts should be made to strengthen the input of public resources such as medical treatment, health, and education in the central and western regions. China should strengthen environmental regulation thresholds, protect the ecological environment, promote equal access to public health services in all regions, improve the ability to prevent and control infectious diseases and the health of residents, so as to achieve healthy and balanced development in all regions.

## Limitation

This article has two limitations: firstly, this paper uses the previous data to discuss the relationship between urbanization and the spread of infectious diseases. It would be better if it could be combined with the latest COVID-19 data. However, the dimensions of urbanization and COVID-19 data are different, so it is impossible to conduct empirical analysis. Therefore, the research of this paper can provide a reference for related research in the future. Secondly, there are many factors affecting the spread of infectious diseases. Only some factors can be controlled in this paper, and there is no way to comprehensively consider the impact of other factors on the results in this paper, such as ecological fallacy, solar radiation, and so on. These are the focus of our next research.

## Data Availability Statement

The original contributions presented in the study are included in the article/supplementary material, further inquiries can be directed to the corresponding author.

## Author Contributions

DY: data curation, conceptualization, methodology, and writing—reviewing and editing. XL: data curation and writing—original draft preparation. JY: visualization and investigation. XS: writing—original draft preparation and investigation. PL: validation and writing—reviewing and editing. PT: writing—reviewing and editing. All authors contributed to the article and approved the submitted version.

## Funding

The authors gratefully acknowledge funding from Research Projects No. 18ZDA038 from the National Social Science Fund of China and No. 20BJL053 from the National Social Science Fund of China.

## Conflict of Interest

The authors declare that the research was conducted in the absence of any commercial or financial relationships that could be construed as a potential conflict of interest.

## Publisher's Note

All claims expressed in this article are solely those of the authors and do not necessarily represent those of their affiliated organizations, or those of the publisher, the editors and the reviewers. Any product that may be evaluated in this article, or claim that may be made by its manufacturer, is not guaranteed or endorsed by the publisher.

## Footnotes

^1^Category B infectious diseases include COVID-19, SARS, AIDS, pertussis, diphtheria, neonatal tetanus, malaria, H7N9 avian influenza and other 27 infectious diseases.

^2^Based on the moment condition, the GMM method constructs equations with parameters. It does not need to assume the distribution of variables, and does not need to know the distribution information of random interference terms, so it can effectively solve the endogenous problem.
